# Obituary

**Published:** 1890-08

**Authors:** 


### Obituary.

EDITOR DENTAL REGISTER, CINCINNATI, O.

*Dear Sir:*—At a meeting of the St. Louis Dental Society, held on Tuesday, May 20th, 1890, the following resolutions on the death of Dr. Homer Judd were adopted:

WHEREAS, The members of the St. Louis Dental Society have learned, with great sorrow, that death has removed from us our loved and honored associate, Dr. Homer Judd, and

WHEREAS, By reason of his great natural abilities, ripe scholarship, zeal, industry and integrity, he was recognized by the dental profession as one of its most influential members; a man who devoted his life to the honor and advancement of his profession, and

WHEREAS, During a long and professional life his relations with this society have been such that it is our pleasure and duty to record our high appreciation of him, therefore,

*Resolved*, That by the death of Dr. Homer Judd, the dental profession has been deprived of one of its most able and useful members; one whose influence for good will live forever.

*Resolved*, That we extend to his family our sincere sympathy in their great bereavement.

*Resolved*, That a copy of these resolutions be sent to the family of the deceased, and to the dental journals for publication.

W. H. EAMES,
HENRY FISHER,
A. J. PROSSER, Committee.

MRS. E. E. CHASE, Corresponding Secretary.

